# A Novel Case and Treatment

**Published:** 1870-04

**Authors:** S. J. Cobb

**Affiliations:** Nashville


### A NOVEL CASE AND TREATMENT.

### THREE ROOTS OF TEETH OR A TOOTH BLOWN OUT AT THE NOSE.

A lady who was so unfortunate as to lose, at the age of twenty, all of her upper teeth, except the second left superior molar, which was broken, leaving the roots, over which she has worn a plate for ten or twelve years, called upon a Dentist, a short time ago, for the purpose of having her plate refitted, and he very naturally suggested the necessity of removing the three loose roots, which she readily consented to; and, in his efforts to remove them, pushed them up into the antrum. The operation becoming a little painful to patient, and frightful to operator, it was no longer persisted in. But, in about eight hours from that time, they were blown out at the nose.

I presume the floor of the antrum covering these roots had been necrosed and partially exfoliated for some time, from the fact that she has since called upon me to operate and treat for diseased antrum. In diagnosing the case, I found from necrosis and exfoliation not only the floor of the antrum covering these roots destroyed, but a portion of the ethmoid and inferior turbinated bones. Making an opening sufficiently large for these roots to pass into the nasal fossa, from which they passed out at the nose. I also found there had been a constant capious fetid discharge from the nose for five years, following an attack of erysipelas of the face.

In operating, I removed all of the necrosed and exfoliated bone, after which I passed well up into the parts a small piece of sponge, thoroughly saturated with two parts carbolic acid and one-third iodine. I then diluted with soft-water the acid and iodine, and syringed the parts well, and sent my patient home, with the direction to use as a wash for the parts the compound acid, iodine and water; also to keep the parts well cleaned with tepid water, and take in the way of general treatment ten grains blue mass, followed by one or two doses citrate of magnesia, after which take three times a day twenty drops syrup of the iodide of iron. For two or three days after the operation, the discharge was slightly increased, as I anticipated, having used the strong solution of acid and iodine for the purpose of producing a sufficient sloughing to bring away such small detached pieces of bone as might remain somewhat attached to the soft parts. In the course of a week, the discharge commenced decreasing rapidly, at which time I fitted a plate and teeth to the jaw, covering the parts well, for the purpose of keeping particles of food and other matter out of the antrum.

At the end of twenty days treatment, the discharge ceased entirely; and, upon examination, the secretions were found to be as healthy as they ever were. S. J. COBB.

NASHVILLE, February, 1870.

